# The novel *RASSF6 *and *RASSF10 *candidate tumour suppressor genes are frequently epigenetically inactivated in childhood leukaemias

**DOI:** 10.1186/1476-4598-8-42

**Published:** 2009-07-01

**Authors:** Luke B Hesson, Thomas L Dunwell, Wendy N Cooper, Daniel Catchpoole, Anna T Brini, Raffaella Chiaramonte, Mike Griffiths, Andrew D Chalmers, Eamonn R Maher, Farida Latif

**Affiliations:** 1 Department of Medical and Molecular Genetics, Department of Reproductive and Child Health, Institute of Biomedical Research, Medical School, University of Birmingham, Edgbaston, B15 2TT, UK; 2 The Children's Hospital at Westmead, Locked Bay 4001, Westmead, NSW, 2145, Australia; 3 Department of Medical Pharmacology, Faculty of Medicine, Università degli Studi di Milano, Italy; 4 Department of Medicine, Surgery and Dentistry, Università degli Studi di Milano, via Di Rudinì 8, 20142 Milan, Italy; 5 West Midlands Regional Genetics Service, Birmingham Women's Hospital, Edgbaston, Birmingham, B15 2TG, UK; 6 Centre for Regenerative Medicine, Department of Biology and Biochemistry, University of Bath, Bath BA2 7AY, UK

## Abstract

**Background:**

The Ras-assocation family (RASSF) of tumour suppressor genes (TSGs) contains 10 members that encode proteins containing Ras-assocation (RA) domains. Several members of the RASSF family are frequently epigenetically inactivated in cancer, however, their role in leukaemia has remained largely uninvestigated. Also, *RASSF10 *is a predicted gene yet to be experimentally verified. Here we cloned, characterised and demonstrated expression of *RASSF10 *in normal human bone marrow. We also determined the methylation status of CpG islands associated with *RASSF1–10 *in a series of childhood acute lymphocytic leukaemias (ALL) and normal blood and bone marrow samples.

**Results:**

COBRA and bisulphite sequencing revealed *RASSF6 *and *RASSF10 *were the only RASSF members with a high frequency of leukaemia-specific methylation. *RASSF6 *was methylated in 94% (48/51) B-ALL and 41% (12/29) T-ALL, whilst *RASSF10 *was methylated in 16% (8/51) B-ALL and 88% (23/26) T-ALL. *RASSF6 *and *RASSF10 *expression inversely correlated with methylation which was restored by treatment with 5-aza-2'deoxycytidine (5azaDC).

**Conclusion:**

This study shows the hypermethylation profile of RASSF genes in leukaemias is distinct from that of solid tumours and represents the first report of inactivation of *RASSF6 *or *RASSF10 *in cancer. These data show epigenetic inactivation of the candidate TSGs *RASSF6 *and *RASSF10 *is an extremely frequent event in the pathogenesis of childhood leukaemia. This study also warrants further investigation of the newly identified RASSF member *RASSF10 *and its potential role in leukaemia.

## Background

Mutation of the Ras superfamily of proteins, or of upstream and downstream signalling components, plays a significant role in the pathogenesis of most forms of cancer. Ras proteins interact with a wide variety of downstream effectors to regulate several signalling pathways important for normal cellular growth [1]. However, Ras proteins can also regulate apoptosis via several recently described effectors, namely the RASSF family of tumour suppressor proteins. The RASSF family consists of six 'classical' members, *RASSF1–6*, that contain C-terminal RA (of the RalGDS/AF-6 variety) and Sav/RASSF/Hippo (SARAH) protein interaction domains. Previously we and others had demonstrated that several RASSF members, namely *RASSF1A*, *RASSF2*, *RASSF4 *and *RASSF5A *are frequently epigenetically inactivated by promoter region CpG island hypermethylation in a broad range of solid tumour types [2,3]. These proteins can regulate apoptosis through several downstream effectors such as the mammalian Serine/Threonine kinases MST1 and MST2 and modulator of apoptosis-1 (MOAP-1) [4-9]. Very recently several additional RA domain-containing family members have been identified and designated *RASSF7 *(also known as HRC1 located 11p15.5), *RASSF8 *(also known as *HOJ-1 *or *C12ORF2 *located 12p12.1), *RASSF9 *(also known as *P-CIP1 *or *PAMCI *located 12q21.31) and *RASSF10 *(also known as *LOC644943 *located 11p15.2). These genes encode proteins that are divergent and structurally distinct from RASSF1–6 and contain an RA domain within their extreme N-termini but lack the SARAH domain. Accordingly, RASSF7–10 are referred to as the 'N-terminal' RASSF family to distinguish them from the 'classical' members RASSF1–6. RASSF7–10 represent an evolutionarily conserved group of proteins with orthologues of all four in the lower vertebrate *Xenopus laevis *and a RASSF7/8 homologue [GenBank:CG5053], a RASSF9 homologue [GenBank:CG13875] and a RASSF10 homologue [GenBank:CG32150] in *Drosophila melanogaster *distinct from the RASSF1–6 *Drosophila *homologue [GenBank:CG4656-PA] [10].

To date the role of the RASSF family in childhood leukaemia has remained uninvestigated and inactivation of the 'N-terminal' RASSF members has not been investigated in any cancer. In this report we describe investigation of the RASSF family for inactivation by promoter CpG island hypermethylation in a large series of childhood B- and T-ALL and find very frequent promoter hypermethylation and loss of expression of *RASSF6 *and *RASSF10 *in B-ALL and T-ALL respectively. These findings support the candidacy of *RASSF6 *and *RASSF10 *as TSGs involved in the pathogenesis of childhood leukaemias.

## Results

### Inactivation of *RASSF6 *in childhood leukaemia

**Figure 1:**
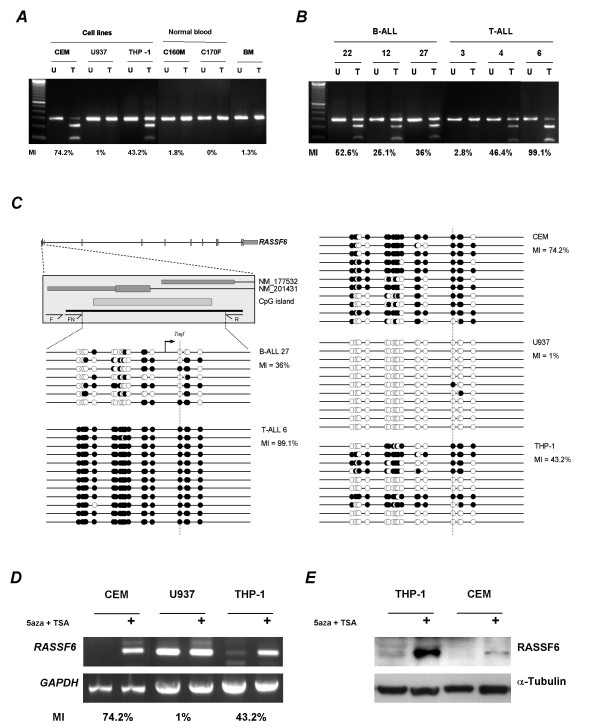
***RASSF6 *is hypermethylated in childhood leukaemias**. ***A***, COBRA analysis showing methylation of *RASSF6 *in leukaemia cell lines. No methylation was observed in normal blood or bone marrow (BM). The methylation index (MI) for each is shown below the gel image. U = undigested PCR product; T = *Taq*I digested PCR product. ***B***, COBRA analysis of B-ALL and T-ALL childhood leukaemias. ***C***, Cloning and bisulphite sequencing of the *RASSF6 *CpG island. Top left shows a schematic of the region analysed relative to the *RASSF6 *gene. Exon 1 of *RASSF6A *[GenBank:NM_177532] and *RASSF6B *[GenBank:NM_201431] is shown relative to the CpG island region (hatched bar), the region amplified by COBRA PCR (black bar) and the location of COBRA primers (F, FN and R). The remaining 5 panels show representative data from primary B-ALL and T-ALL leukaemias 27 and 6 respectively with leukaemia cell lines CEM, U937 and THP-1. Each horizontal line represents an individual allele whilst the circles represent single CpG dinucleotides. Filled circle represents a methylated CpG dinucleotide whereas an open circle represents an unmethylated CpG dinucleotide. Indicated by the arrow is the transcription start site of *RASSF6A*. The dashed vertical line indicates the CpG dinucleotide within the *Taq*I restriction site (TCGA) used to assay for methylation. ***D***, Methylation of the *RASSF6 *CpG island correlates with loss or downregulation of *RASSF6 *expression. Methylated cell lines CEM, THP-1 and the unmethylated cell line U937 were cultured in the presence or absence of 5azaDC and TSA. RT-PCR analysis showed loss or downregulation of *RASSF6 *expression correlates with the methylation status of the *RASSF6 *CpG island (***A ***and ***C***). *RASSF6 *expression is restored following 5azaDC and TSA treatment. *GAPDH *was used as a control for RNA integrity and equal loading. ***E***, RASSF6 protein expression is lost or downregulated in methylated leukaemia cell lines. We used an antibody towards human RASSF6 protein to investigate expression before and after 5azaDC and TSA treatment. α-Tubulin was used as a control for equal loading.

We investigated the CpG island methylation status of the 'classical' RASSF members, *RASSF1–6*, in a large series of childhood ALL to ascertain whether epigenetic inactivation of these genes occurs in childhood leukaemia. Surprisingly, we found that *RASSF1–5 *were infrequently methylated whilst *RASSF6 *was methylated in B-ALL (94%; 48/51), T-ALL (41%; 12/29) and 5/5 unclassified childhood leukaemias but not in normal blood or bone marrow control samples (0/8 and 0/1 respectively; figure 1A, B, table 1 and Additional file 1; figure S1–2). Given that *RASSF6 *epigenetic inactivation has not previously been observed in any cancer we next sought to determine the extent of methylation across the *RASSF6 *CpG island and determine whether methylation correlated with loss or downregulation of *RASSF6 *expression. Bisulphite sequencing of up to 12 individual alleles from 9 leukaemia cell lines, 6 B-ALL, 6 T-ALL, 1 pre-B-ALL, 6 normal blood and 1 normal bone marrow sample confirmed leukaemia-specific methylation across the entire *RASSF6 *CpG island in cell lines, B-ALL and T-ALL (figure 1C, and Additional file 1; figure S3 and Additional file 2; table S1). Whilst RT-PCR showed *RASSF6 *was expressed in all normal tissues analysed including bone marrow (see Additional file 1; figure S4A), and in the unmethylated leukaemia cell line U937 (figure 1D and Additional file 1; figure S4B), we observed absent or downregulated expression of *RASSF6 *in methylated leukaemia cell lines. Treatment of hypermethylated leukaemia cell lines with 5azaDC and TSA (Trichostatin A) reactivated *RASSF6 *expression (figure 1D and Additional file 1; figure S4B). Using a commercially available antibody that recognises human RASSF6 protein we also observed restoration of RASSF6 protein expression following 5azaDC and TSA treatment in protein lysates from leukaemia lines (figure 1E). Thus methylation of the *RASSF6 *CpG island results in loss of *RASSF6 *expression. As mentioned previously, several RASSF members have been shown to bind Ras proteins, including RASSF6 [11]. Previously we had also demonstrated that inactivation of *RASSF2 *by promoter hypermethylation inversely correlates with K-Ras mutation status in colorectal carcinomas [12]. We investigated our series of leukaemias for mutations to codons 12, 13 and 61 of both K- and N-Ras. K-Ras mutations were found in 2.2% (2/89) whilst N-Ras mutations were found in 11.2% (10/89) leukaemias. However, no association was found between Ras mutation status and *RASSF6 *methylation status.

**Table 1:** A summary of *RASSF6 *and *RASSF10 *promoter methylation in leukaemia and control samples.

**TISSUE/TUMOUR TYPE**	*RASSF6*	*RASSF10*
Leukaemia cell lines	**10/11(91%)**	**7/7(100%)**

Childhood B-ALL	**48/51(94%)**	**8/51(16%)**

Childhood T-ALL	**12/29(41%)**	**23/26(88%)**

Childhood Pre-B-ALL	0/1	0/1

Unclassified Childhood Leukaemia	**5/5**	0/5

Normal Blood	0/8	0/21

Normal Bone Marrow	0/1	0/1

### Characterisation of the human *RASSF10 *gene

**Figure 2:**
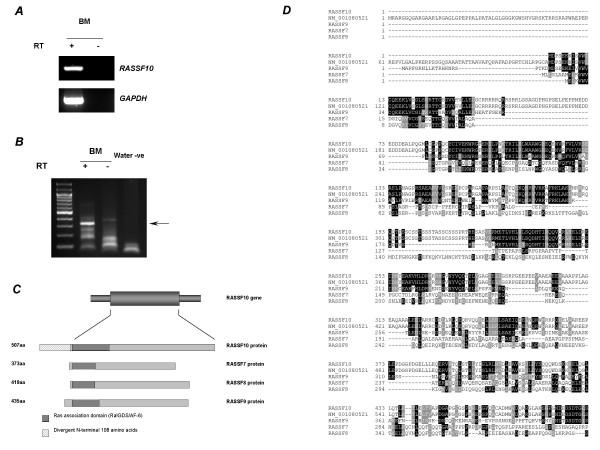
**Characterisation of the *RASSF10 *gene**. ***A***, RT-PCR analysis showing expression of *RASSF10 *in normal human bone marrow. ***B***, The 5' region of the *RASSF10 *transcript was amplified from normal human bone marrow using 5'RACE. Arrowed is the major product of 5'RACE which was cloned and sequenced to determine the transcription start point and open reading frame of the *RASSF10 *gene. ***C***, Schematic summarising the structure of the *RASSF10 *gene and the open reading frame encoding a shortened protein of 507amino acids that does not contain the *in silico *predicted N-terminal 108 amino acids that are divergent from the RASSF7–9 proteins. ***D***, ClustalW alignments of RASSF7–10 proteins including both the *in silico *predicted 615 amino acid RASSF10 [GenBank:NM_001080521] and the shorter 507 amino acid RASSF10 confirmed in this study suggesting the divergent N-terminal 108 amino acids is not likely to be part of the RASSF10 protein.

The human *RASSF10 *gene has been predicted based on homology with *RASSF9/P-CIP1 *but has yet to be supported by experimental evidence. UCSC  and Ensembl  human genome browsers predict a 615 amino acid protein encoded by a two exon gene separated by a short intron of 104 bp. Interestingly, no 5' or 3'UTRs were predicted, hence we reasoned *RASSF10 *had not been completely characterised. Sequencing of the only IMAGE clone available [IMAGE:4413366] [GenBank:BG034782] obtained from a human liver cDNA library revealed a 3'UTR of 478 bp beyond the predicted stop codon followed by a poly A-tail. Contrary to the UCSC and Ensembl genome browsers however, the IMAGE clone sequence suggested the *RASSF10 *gene did not contain introns and predicted alternative transcription and translation initiation sites located Chr11:12,987,466 and Chr11:12,987,700 respectively (UCSC genome browser, March 2006 freeze). Analysis of the sequence flanking this alternative ATG (GCC**ATG**G) support its candidacy as a genuine translation initiation site since it matches the consensus 'Kozak' sequence of A/GCC**ATG**G in which the important -3 and +4 A/G and G residues respectively (underlined) were conserved [13,14]. Furthermore, translation initiation site prediction software at  also predicted the ATG at Chr11:12,987,700 is the translation initiation site within the *RASSF10 *gene. This suggested the *RASSF10 *gene contains a shorter open reading frame encoding a 507 amino acid protein. However, since the IMAGE clone may be incomplete we confirmed the transcription start site of the *RASSF10 *gene using 5'RACE. We first demonstrated expression of the *RASSF10 *transcript in *DNaseI*-treated normal human bone marrow RNA using RT-PCR (figure 2A). This RNA was then subjected to 5'RACE using *RASSF10*-specific antisense primers, which generated a major product of ~330 bp and several larger and smaller minor products (figure 2B). These products were gel purified, cloned and sequenced to reveal a transcription start site located at Chr11:12,987,444 (UCSC genome browser, March 2006 freeze) with two alternative transcription start sites at +119 bp and +169 bp (Chr11:12,987,565 and Chr11:12,987,613, respectively). This revealed the presence of a 5'UTR of up to 431 bp and confirms the *RASSF10 *gene likely encodes a much shorter 507 amino acid protein. That the predicted N-terminal 108 amino acids may not actually be part of the RASSF10 protein is supported by ClustalW alignments of RASSF7–10. These alignments show the predicted N-terminal 108 amino acids are extremely divergent with no significant homology to RASSF7–9 or with any other human protein (figure 2C and 2D). Interestingly, the Rassf10 murine protein [Genbank:NP_780488] also lacks this divergent N-terminus and is 508 amino acids in length. Therefore, we have characterised the human *RASSF10 *gene and verified expression in normal bone marrow. We provide evidence that *RASSF10 *is a single exon gene with a 5'UTR of up to 431 bp, a 3'UTR of 478 bp and an open reading frame encoding a protein of 507 amino acids, not 615 amino acids as predicted by current versions of human genome browsers.

### Inactivation of *RASSF10 *in childhood leukaemia

**Figure 3:**
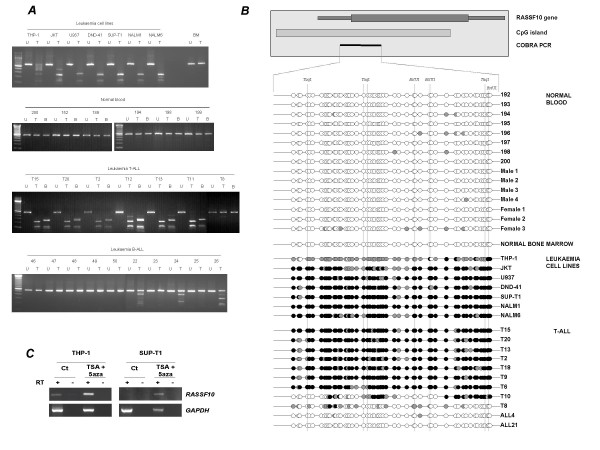
***RASSF10 *is hypermethylated in childhood leukaemias**. ***A***, COBRA analysis showing methylation status of *RASSF10 *in leukaemia cell lines and normal bone marrow, normal blood, primary T-ALL and primary B-ALL. No methylation was observed in normal blood or bone marrow (BM). U = undigested PCR product; T = *Taq*I digested PCR product. B = *BstU*I digested PCR product. ***B***, Direct bisulphite sequencing of normal blood, normal bone marrow, leukaemia cell lines and primary T-ALL leukaemias. Top shows a schematic of the region amplified by COBRA PCR (black bar) relative to the *RASSF10 *gene and CpG island region (hatched bar). Each horizontal line represents the total DNA amplified from the sample whilst the circles represent single CpG dinucleotides. Filled black circle represents a completely methylated CpG dinucleotide, open circle represents a completely unmethylated CpG dinucleotide and filled gray circle represents a partially methylated CpG dinucleotide. The dashed vertical line indicates the CpG dinucleotides within *Taq*I (TCGA) and *Bst*UI (CGCG) restriction sites used to assay for methylation. ***C***, Methylation of the *RASSF10 *CpG island correlates with loss or downregulation of *RASSF10 *expression. The partially methylated cell line THP-1 and the completely methylated cell line SUP-T1 were cultured in the presence or absence of 5azaDC and TSA. RT-PCR analysis showed loss or downregulation of *RASSF10 *expression correlates with the methylation status of the *RASSF10 *CpG island (***A ***and ***B***). *RASSF10 *expression is restored following 5azaDC and TSA treatment. *GAPDH *was used as a control for RNA integrity and equal loading. Ct = control mock treated; RT = reverse transcriptase.

*RASSF10 *contains a large CpG island that covers the majority of the gene. Given the expression of *RASSF10 *in bone marrow we questioned whether *RASSF10 *is also inactivated by promoter methylation in the same series of childhood leukaemias. We investigated the *RASSF10 *CpG island for DNA hypermethylation using COBRA and bisulphite sequencing. The *RASSF10 *CpG island was heavily methylated in 7/7 leukaemia cell lines (figure 3A). Next, we investigated childhood B- and T-ALL, normal blood and normal bone marrow for evidence of *RASSF10 *CpG island hypermethylation. We found *RASSF10 *was methylated in 23/26 (88%) T-ALL and 8/51 (16%) B-ALL but was unmethylated in normal bone marrow and 21 normal blood samples (figure 3A, table 1). Direct bisulphite sequencing of the *RASSF10 *CpG island confirmed the presence of dense methylation across the entire region analysed (figure 3B). To investigate the effects of methylation on *RASSF10 *expression we analysed the leukaemia cell lines THP-1 and SUP-T1 using RT-PCR. We found low level expression of *RASSF10 *in THP-1 (partially methylated) and loss of *RASSF10 *expression in SUP-T1 (completely methylated) and in both cases expression was upregulated following 5azaDC and TSA treatment (figure 3C). Thus, methylation of the *RASSF10 *CpG island inactivates expression of the gene very frequently in T-ALL and in a subset of B-ALL.

Next, we investigated the remaining 'N-terminal' RASSF members to determine whether they too become inactivated in childhood leukaemias. *RASSF7 *and *RASSF8 *both contain 5' CpG islands encompassing their first exon, whilst *RASSF9 *does not contain a CpG island. As determined by COBRA and bisulphite sequencing, *RASSF7 *was unmethylated in all leukaemia cell lines analysed (0/5). *RASSF8 *methylation was found to be infrequent, occurring in 2/6 leukaemia cell lines, 2/19 (10%) childhood T-ALLs, and 4/46 (9%) B-ALL with no methylation detectable in normal bone marrow or normal blood (0/7). Methylation of the *RASSF8 *CpG island correlated with loss of *RASSF8 *expression which was restored following treatment with 5azaDC (and Additional file 1; figure S5).

## Discussion

In recent years several members of the classical RASSF family have emerged as important TSGs [2,3,15]. Whether inactivation of *RASSF1*-*6 *plays a role in leukaemia has not yet been determined. We found that *RASSF1*-*5 *were not frequently methylated in our series of childhood ALL. Instead, we observed very frequent inactivation of *RASSF6 *reiterating the importance of several classical RASSF members in the development of different forms of cancer. Evidence is accumulating that *RASSF6 *plays a role in tumourigenesis and likely functions as a regulator of apoptosis [2]. *RASSF6 *is downregulated in 30–60% of a variety of solid tumours [11]. SiRNA-mediated knockdown of *RASSF6 *enhances tumourigenic growth in soft agar whilst *RASSF6 *overexpression reduces tumour growth in a variety of tumour cell lines [11,16]. The growth inhibitory properties of *RASSF6 *are most likely a result of induction of apoptosis since *RASSF6*-induced cell death was concomitant with a variety of apoptotic markers [11,16]. Previously, we had demonstrated that *RASSF6 *interacted with K-Ras in a GTP-dependent manner and that *RASSF6*-induced apoptosis was enhanced synergistically by co-transfection with constitutively active K-RasG12V [11]. Thus, *RASSF6 *can mediate the growth inhibitory properties of Ras proteins. In agreement with previous studies [17-19] the frequency of mutations to codons 12, 13 and 61 of both K- and N-Ras combined in our series of leukaemias was only 14% (12/89). Therefore, inactivation of *RASSF6 *may provide the alternative mechanism of Ras pathway inactivation in B-ALL. In T-ALL, in which *RASSF6 *methylation is less frequent, *RASSF1A*, *RASSF5A *and *RASSF6 *methylation may account for Ras pathway inactivation in the majority of cases.

The recently identified 'N-terminal' RASSFs consist of four members designated *RASSF7–10 *[20]. *RASSF7–10 *represents a structurally distinct yet evolutionarily conserved sub-group of the RASSF family. It has not yet been determined whether RASSF7–10 bind to Ras proteins. We found no evidence of methylation of *RASSF7 *in leukaemia cell lines. Hypermethylation of the *RASSF8 *CpG island and loss of *RASSF8 *expression was observed in 2/6 leukaemia cell lines.

However, since we observed *RASSF8 *CpG island hypermethylation in only 2/19 T-ALLs and 4/46 B-ALLs we suspect inactivation of *RASSF8 *may be involved in the pathogenesis of only a small subset of leukaemias. *RASSF9 *was not investigated for inactivation by DNA methylation due to lack of a CpG island.

*LOC644943 *was very recently designated *RASSF10 *based on its homology with *RASSF9/P-CIP1*. The existence of this gene in humans has yet to be experimentally verified. However, the *Drosophila *homologue, *CG32150*, is expressed in several sensory organ precursors and seems to be required for correct Hedgehog pathway signaling [21,22]. Orthologues of human *RASSF10 *can be found in several model species including *X.laevis *[GenBank:NM_001115020] and *M.musculus *[GenBank:NM_175279]. Murine *Rassf10 *is expressed in several tissues including salivary gland, testes, kidney, lung and brain. *X.laevis *and *M.musculus Rassf10 *proteins share 60 and 85% identity with human *RASSF10 *respectively. To date there has been no investigation of vertebrate *RASSF10*. Here we characterized the human *RASSF10 *gene and transcript and demonstrate expression in normal human bone marrow. Whether *RASSF10 *is expressed in other tissues was not determined in the present study, though this is likely since we obtained an IMAGE clone derived from a normal human liver cDNA library. Furthermore, *in silico *sources (Unigene [Hs.693473] and Genecards) provide further evidence of expression in brain, skeletal muscle, pancreas, liver and trachea. Interestingly, we demonstrate that current genome browser predictions of the *RASSF10 *gene and protein may require revision and provide evidence of alternative transcription and translation inititation sites that produce a much shorter protein of 507 amino acids. We also found *RASSF10 *was very frequently inactivated in T-ALL (88%). *RASSF10 *CpG island methylation correlated with loss of *RASSF10 *expression which was restored following 5azaDC and TSA treatment. It is particularly interesting that *RASSF6 *methylation occurs very frequently in B-ALL whilst *RASSF10 *methylation is more restricted to T-ALL. It is unclear at present whether this represents independent routes to inactivation of the same pathway or whether it reflects inactivation of separate pathways important in the development of these different subtypes of leukaemia. Nevertheless, inactivation of *RASSF6 *or *RASSF10 *by promoter DNA hypermethylation appears to be an event associated with the majority of childhood leukaemias.

## Conclusion

In summary, we show that the hypermethylation profile of RASSF genes in leukaemias is distinct from that of solid tumours and show for the first time that *RASSF6 *is also inactivated in cancer, but specifically in leukaemias. Given the emerging role of *RASSF6 *as a Ras effector and possible TSG involved in the regulation of apoptosis it is likely that loss of expression may be involved in the ability of leukaemic cells to evade apoptosis. Our data also suggests that of the newly identified 'N-terminal' RASSF genes, *RASSF10 *is an interesting candidate for further analysis in other types of cancer and for functional investigation to ascertain its role in leukaemias.

## Methods

### Tumour samples

Twelve leukaemia cell lines (DND-41, CCRF-CEM (CEM), U937, Jurkat (JKT), TALL-1, NALM1, NALM6, NALM16, NALM17, THP-1, SUP-T1 and MOLT-4) and 89 primary childhood ALL comprising 51 B-cell ALL (B-ALL), 32 T-cell ALL (T-ALL), 1 pre-B-ALL and 5 unclassified leukaemias were analysed. In addition a total of 21 normal blood samples and 1 normal bone marrow (BM, AMS Biotechnology) sample were used as controls. All DNA samples were obtained with informed consent from patients and family members.

### Methylation analysis

Bisulphite modification was performed as described previously [23]. Promoter methylation status of *RASSF1A *and *RASSF2 *were determined using MSP as described previously [12]. The methylation status of *RASSF3*, *RASSF4 *and *RASSF5A *were determined by combined bisulphite restriction analysis (COBRA) as described in Hesson *et al*., [12], Hesson *et al*., [23] and Eckfeld *et al*., [24]. For *RASSF6*, primers were designed to amplify the entire CpG island encompassing the first exons of *RASSF6A *(NM_177532) and *RASSF6B *(NM_201431) from bisulphite modified DNA using semi-nested bisulphite PCR (see additional file 2; table S2 for primer sequences). The methylation status of *RASSF7*, *RASSF8 *and *RASSF10 *were determined by COBRA and direct bisulphite sequencing. For *RASSF7 *and *RASSF8 *primers were designed to amplify a portion of the CpG island encompassing exon 1. The *RASSF10 *CpG island covers the majority of the gene. We designed primers to amplify a portion of the CpG island encompassing the 5' region of the gene. Five microlitres of PCR product was incubated with 2 U *TaqI *restriction enzyme (TCGA) for 2 h at 65°C or 2 U *BstUI *restriction enzyme (CGCG) for 2 h at 60°C before visualisation on a 2% agarose gel. Samples selected for bisulphite sequencing were cloned (*RASSF6*) into the CloneJET vector (Fermentas) according to manufacturers instructions or sequenced directly (*RASSF8 *and *RASSF10*). Up to 12 individual colonies were chosen for colony PCR using the *RASSF6 *COBRA primers. Products were then sequenced to ascertain the methylation status of individual alleles and to determine the methylation index (MI). The MI was calculated as a percentage using the equation; number of CpG dinucleotides methylated/total number of CpG dinucleotides sequenced × 100.

### Cell lines, 5azaDC treatment and RT-PCR

Leukaemia cell lines were maintained in RPMI1640 (Sigma) supplemented with 10% FCS, 2 mM Glutamine, 20 mM HEPES, 1 mM Sodium Pyruvate and 12.6 mM Glucose Monohydrate at 37°C, 5% CO_2_. Cells were treated with 5 μM of the DNA demethylating agent 5azaDC (Sigma) freshly prepared in ddH_2_O and filter-sterilised. The medium (including 5 μM 5azaDC) was changed every day for 5 days. Cells were also treated on day 4 with 0.1 μM TSA for 24 hrs. RNA was prepared using RNA bee (AMS biotechnology) according to manufacturers' instructions. cDNA was generated from 1 μg total RNA using SuperScript III (Invitrogen) and polyN primers. We analysed expression of *RASSF6 *using primers specific for exons 2 and 4 that amplify both the *A *and *B *isoforms (F = 5'-ATGATGGCTCACCAGTACCC-3' and R = 5'-GGTCGTTTTACTCCCCAGAA-3'). We analysed expression of *RASSF10 *using the primers 5'-CCATGACCCAGGAGAAACAG-3' (F) and 5'-TGCTGGCGAATTGTGTGGTC-3' (R). Since *RASSF10 *is a single exon gene we first *DNaseI *(Fermentas) treated all RNA samples prior to cDNA synthesis and included control experiments in which reverse transcriptase was omitted (No RT controls) in all samples analysed. We analysed expression of *RASSF8 *using the primers F = 5'-TCCATTGAGAAACAGCTGGA-3' and R = 5'-TGGCACAAATCAAAAAGGAA-3'. In all cases a *GAPDH *control was included using conditions described previously [12]. *RASSF6, RASSF8 *and *RASSF10 *were amplified from 50 ng cDNA using 0.8 μM of each primer, 3 mM MgCl_2_, 0.25 mM dNTPs and 1 U Fast start *Taq *(Roche).

### Protein expression

Leukaemia lines THP-1 and CEM were treated with 5azaDC as above. Protein extracts were then prepared by lysis in 50 mM TrisHCl pH7.5, 1 mM EDTA, 1 mM EGTA, 50 mM Sodium Fluoride, 5 mM Sodium Pyrophosphate, 1 mM Sodium Orthovanadate, 0.27 M Sucrose, 1% Triton and protease inhibitor cocktail (Roche). Twenty micrograms of protein extracts were resolved by SDS-PAGE, transferred to PVDF membrane and probed for the presence of RASSF6 using 1 μg/mL anti-RASSF6 rabbit-raised polyclonal antibody (ProteinTech Group).

### 5' Rapid Amplification of cDNA Ends (RACE)

One microgram of total RNA from bone marrow (AMS Biotechnology) was *DNaseI *(Fermentas) treated according to manufacturers instructions. We synthesised cDNA using a 5'/3' RACE kit (2^nd ^generation, Roche) and the *RASSF10 *antisense primer 5'-TGCTGGCGAATTGTGTGGTC-3'. The 5' UTR of the *RASSF10 *transcript was obtained using nested PCR with the nested antisense *RASSF10*-specific primers 5'-GCTGTTTCTCCTGGGTCATG-3' and 5'-TATCTTCTTTTCCGAAGGATCCATG-3' used in combination with a polyT-anchor primer. Control experiments in which reverse transcriptase was omitted were also performed to eliminate DNA contamination. PCR products were gel extracted, sequenced and the sequences obtained aligned against the human genome browser.

### Mutation analysis

PCR primers were used to amplify exons 2 and 3 of the K- and N-Ras genes from genomic DNA. The primers were as follows: K-Ras exon 2 F 5'-TTTGTATTAAAAGGTACTGGTGGAG-3'; K-Ras exon 2 R 5'-CCTTTATCTGTATCAAAGAATGGTC-3'; K-Ras exon 3 F 5'-CTGTGTTTCTCCCTTCTCAGG-3'; K-Ras exon 3 R 5'-AGAAAGCCCTCCCCAGTCCT-3'; N-Ras exon 2 F 5'-GATGTGGCTCGCCAATTAAC-3'; N-Ras exon 2 R 5'-GAATATGGGTAAAGATGATCCGAC-3'; N-Ras exon 3 F 5'-GTTAGATGCTTATTTAACCTTGGC-3'; N-Ras exon 3 R 5'-TGTGGTAACCTCATTTCCCC-3'. PCR products were directly sequenced using ABI BigDye cycle sequencing kit (Perkin-Elmer).

## Abbreviations

RASSF: Ras-association family; TSG: tumour suppressor gene; RA: Ras-association domain; ALL: Acute Lymphocytic Leukaemia; 5azaDC: 5-aza-2'deoxycytidine; TSA: Trichostatin A; SARAH: Sav/RASSF/Hpo domain; MSP: Methylation-specific PCR; COBRA: Combined Bisulphite Restriction Analysis; RACE: Rapid Amplification of cDNA Ends.

## Competing interests

The authors declare that they have no competing interests.

## Authors' contributions

LBH acquired the majority of data (involving tissue culture, assay design, molecular genetic studies, bioinformatics and protein analysis), wrote the manuscript and contributed to the design and concept of the study. TLD performed bisulphite modification and contributed to COBRA analysis and mutation screening. WNC performed COBRA, bisulphite sequencing and expression analysis pertaining to RASSF8. DC, MG, RC and ATB provided leukaemia DNA samples and cell lines. ADC and ERM contributed to the concept of the study. FL conceived the studies, oversaw the experimental work and helped draft the manuscript and established all the collaborations. All authors have read and approved the final manuscript.

## Supplementary Material

Additional file 1 **RASSF methylation profile**. The data provided represent analysis of RASSF members and additional data relating to *RASSF6 *and *RASSF10 *in childhood leukaemia. **Figure S1: RASSF methylation profile of leukaemia cell lines**. The methylation status of *RASSF1A *and *RASSF2 *was determined using MSP whereas the methylation status of *RASSF3*-*6 *were determined using COBRA. *RASSF1A*, *RASSF5A *and *RASSF6 *were hypermethylated in leukaemia cell lines whereas *RASSF2 *and *RASSF3 *showed no evidence of methylation. *RASSF4 *was methylated in both leukaemia cell lines and normal blood. For MSP assays M = methylated specific PCR; U = unmethylated specific PCR. For COBRA assays U = undigested PCR product; T = *Taq*I digested PCR product. **Figure S2: RASSF methylation profile of primary leukaemias**. RASSF genes showing evidence of hypermethylation in leukaemia cell lines were investigated for methylation in B-ALL and T-ALL leukaemias. *RASSF1A *and *RASSF5A *were infrequently methylated in T-ALL (2/12 and 2/24 respectively). *RASSF4 *was methylated in 25/25 T-ALL but in only 7/50 B-ALL leukaemias, however given the low level methylation of *RASSF4 *observed in normal blood this gene was not investigated further. *RASSF6 *was methylated in 48/51 (94%) B-ALL and 12/29 (41%) T-ALL but not in normal bone marrow (BM) or blood control samples. For MSP assays M = methylated specific PCR; U = unmethylated specific PCR. For COBRA assays U = undigested PCR product; T = *Taq*I digested PCR product. **Figure S3: Bisulphite sequencing of the *RASSF6 *CpG island**. As in figure 1C up to 12 alleles from B-ALL, T-ALL leukaemia cell lines, normal blood and normal bone marrow were cloned and sequenced. The methylation index (MI) is given for each. **Figure S4: *RASSF6 *expression in normal tissues and leukaemia cell lines**. ***A***, *RASSF6 *mRNA was detected in all tissues analysed. ***B***, Restoration of *RASSF6 *mRNA expression in methylated leukaemia cell lines following treatment with 5azaDC and TSA showing the additional cell lines JKT and REH. *GAPDH *was used as a control for equal loading. **Figure S5: Methylation of the *RASSF8 *CpG island infrequently inactivates *RASSF8 *in leukaemias**. ***A***, Direct bisulphite sequencing of *RASSF8 *CpG island PCR products. Filled black circle represents a completely methylated CpG dinucleotide, open circle represents a completely unmethylated CpG dinucleotide and filled gray circle represents a partially methylated CpG dinucleotide. ***B***, Methylation of the *RASSF8 *CpG island correlates with loss or downregulation of *RASSF8 *expression which is restored following 5azaDC treatment. Click here for file

Additional file 2 **RASSF6 methylation indexes and PCR primers used within this study**. The data provided summarise RASSF6 methylation index data in cell lines, primary tumour and control samples (Table S1). PCR primers used in this study are also listed (Table S2). **Table S1: **A summary of the methylation index of the *RASSF6 *CpG island in leukaemia cell lines, B-ALL, T-ALL and normal blood and bone marrow control samples. **Table S2: **MSP and COBRA primer sequences used within this study. Click here for file
