# Supplementary material for: The novel RASSF6 and RASSF10 candidate tumour suppressor genes are frequently epigenetically inactivated in childhood leukaemias
Source: Mol Cancer. 2009 Jul 1;8:42. doi: 10.1186/1476-4598-8-42 (PMC2711046; doi:10.1186/1476-4598-8-42)
Supplement: Additional file 1 — RASSF methylation profile. The data provided represent analysis of RASSF members and additional data relating to RASSF6 and RASSF10 in childhood leukaemia. Figure S1: RASSF methylation profile of leukaemia cell lines. The methylation status of RASSF1A and RASSF2 was determined using MSP whereas the methylation status of RASSF3-6 were determined using COBRA. RASSF1A, RASSF5A and RASSF6 were hypermethylated in leukaemia cell lines whereas RASSF2 and RASSF3 showed no evidence of methylation. RASSF4 was methylated in both leukaemia cell lines and normal blood. For MSP assays M = methylated specific PCR; U = unmethylated specific PCR. For COBRA assays U = undigested PCR product; T = TaqI digested PCR product. Figure S2: RASSF methylation profile of primary leukaemias. RASSF genes showing evidence of hypermethylation in leukaemia cell lines were investigated for methylation in B-ALL and T-ALL leukaemias. RASSF1A and RASSF5A were infrequently methylated in T-ALL (2/12 and 2/24 respectively). RASSF4 was methylated in 25/25 T-ALL but in only 7/50 B-ALL leukaemias, however given the low level methylation of RASSF4 observed in normal blood this gene was not investigated further. RASSF6 was methylated in 48/51 (94%) B-ALL and 12/29 (41%) T-ALL but not in normal bone marrow (BM) or blood control samples. For MSP assays M = methylated specific PCR; U = unmethylated specific PCR. For COBRA assays U = undigested PCR product; T = TaqI digested PCR product. Figure S3: Bisulphite sequencing of the RASSF6 CpG island. As in figure 1C up to 12 alleles from B-ALL, T-ALL leukaemia cell lines, normal blood and normal bone marrow were cloned and sequenced. The methylation index (MI) is given for each. Figure S4: RASSF6 expression in normal tissues and leukaemia cell lines. A, RASSF6 mRNA was detected in all tissues analysed. B, Restoration of RASSF6 mRNA expression in methylated leukaemia cell lines following treatment with 5azaDC and TSA showing the additional cell lines JKT and REH. GAPDH [file 1476-4598-8-42-S1.ppt]

## Slide 1
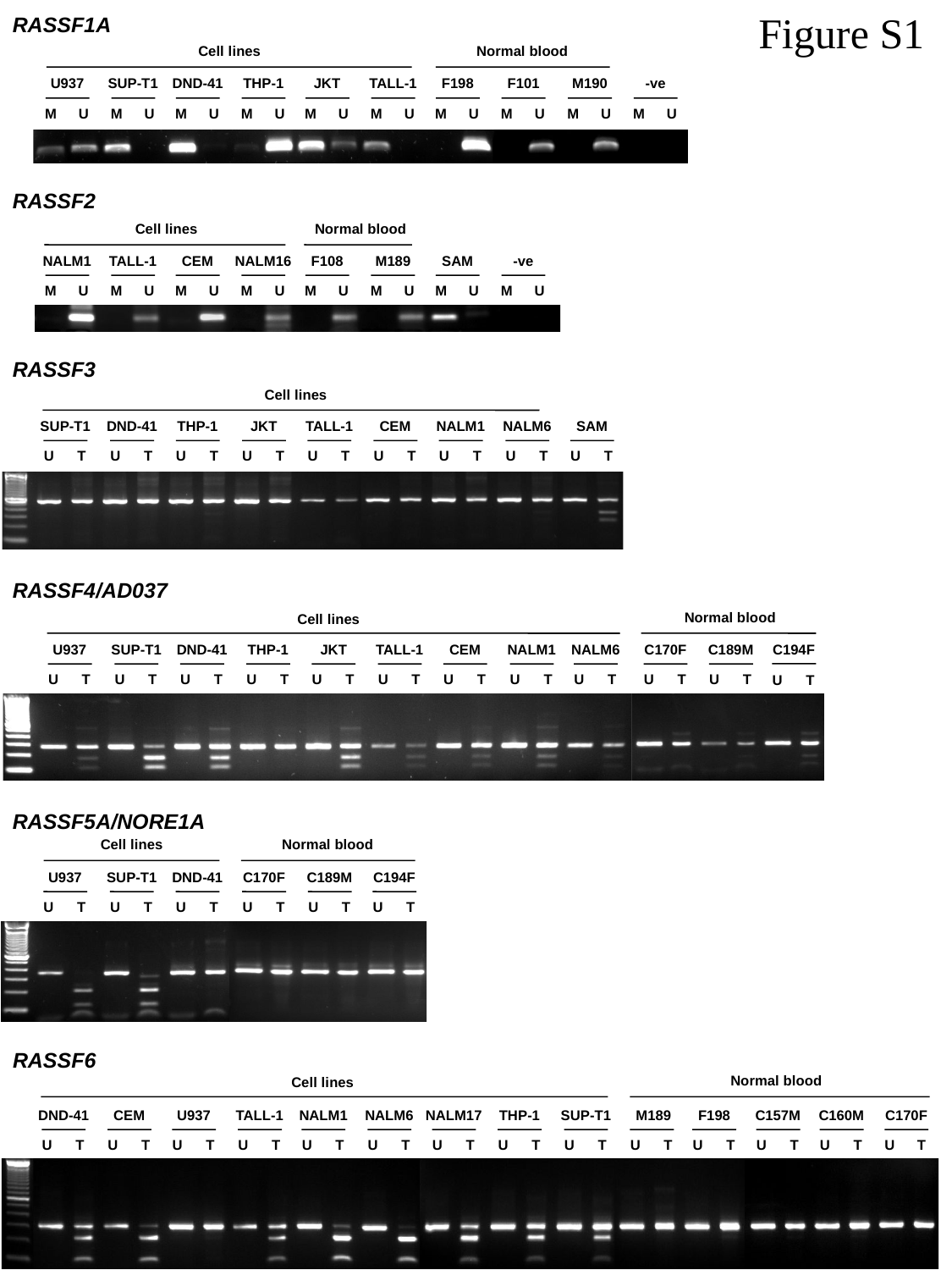

Figure S1
RASSF1A
Cell lines
Normal blood
U937
SUP-T1
DND-41
THP-1
JKT
TALL-1
F198
F101
M190
-ve
M
U
M
U
M
U
M
U
M
U
M
U
M
U
M
U
M
U
M
U
RASSF2
Cell lines
Normal blood
NALM1
TALL-1
CEM
NALM16
F108
M189
SAM
-ve
M
U
M
U
M
U
M
U
M
U
M
U
M
U
M
U
RASSF3
Cell lines
SUP-T1
DND-41
THP-1
JKT
TALL-1
CEM
NALM1
NALM6
SAM
U
T
U
T
U
T
U
T
U
T
U
T
U
T
U
T
U
T
RASSF4/AD037
Normal blood
Cell lines
U937
SUP-T1
DND-41
THP-1
JKT
TALL-1
CEM
NALM1
NALM6
C170F
C189M
C194F
U
T
U
T
U
T
U
T
U
T
U
T
U
T
U
T
U
T
U
T
U
T
U
T
RASSF5A/NORE1A
Cell lines
Normal blood
U937
SUP-T1
DND-41
C170F
C189M
C194F
U
T
U
T
U
T
U
T
U
T
U
T
RASSF6
Normal blood
Cell lines
DND-41
CEM
U937
TALL-1
NALM1
NALM6
NALM17
THP-1
SUP-T1
M189
F198
C157M
C160M
C170F
U
T
U
T
U
T
U
T
U
T
U
T
U
T
U
T
U
T
U
T
U
T
U
T
U
T
U
T

## Slide 2
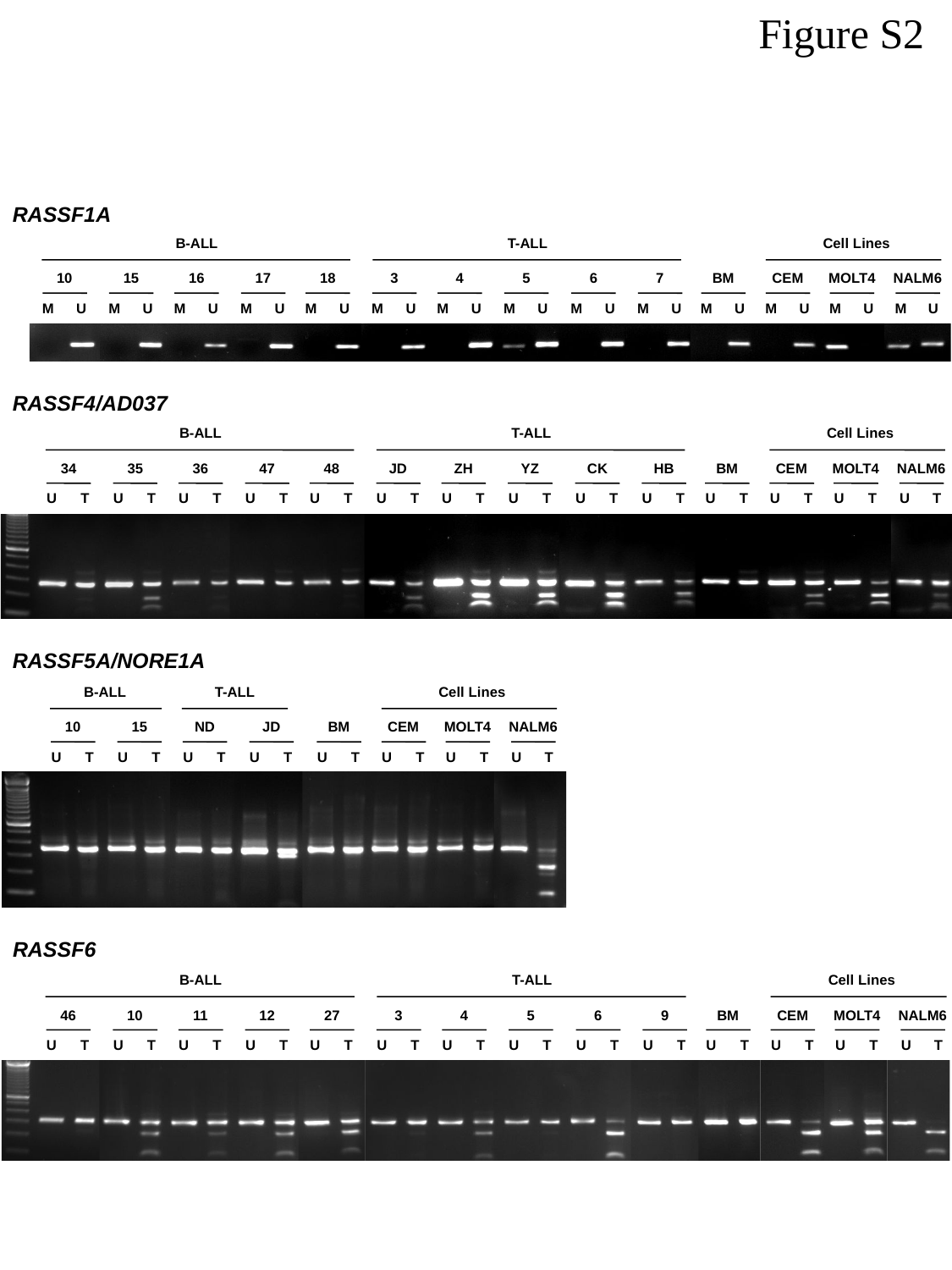

Figure S2
RASSF1A
B-ALL
T-ALL
Cell Lines
10
15
16
17
18
3
4
5
6
7
BM
CEM
MOLT4
NALM6
M
U
M
U
M
U
M
U
M
U
M
U
M
U
M
U
M
U
M
U
M
U
M
U
M
U
M
U
RASSF4/AD037
B-ALL
T-ALL
Cell Lines
34
35
36
47
48
JD
ZH
YZ
CK
HB
BM
CEM
MOLT4
NALM6
U
T
U
T
U
T
U
T
U
T
U
T
U
T
U
T
U
T
U
T
U
T
U
T
U
T
U
T
RASSF5A/NORE1A
B-ALL
T-ALL
Cell Lines
10
15
ND
JD
BM
CEM
MOLT4
NALM6
U
T
U
T
U
T
U
T
U
T
U
T
U
T
U
T
RASSF6
B-ALL
T-ALL
Cell Lines
46
10
11
12
27
3
4
5
6
9
BM
CEM
MOLT4
NALM6
U
T
U
T
U
T
U
T
U
T
U
T
U
T
U
T
U
T
U
T
U
T
U
T
U
T
U
T

## Slide 3
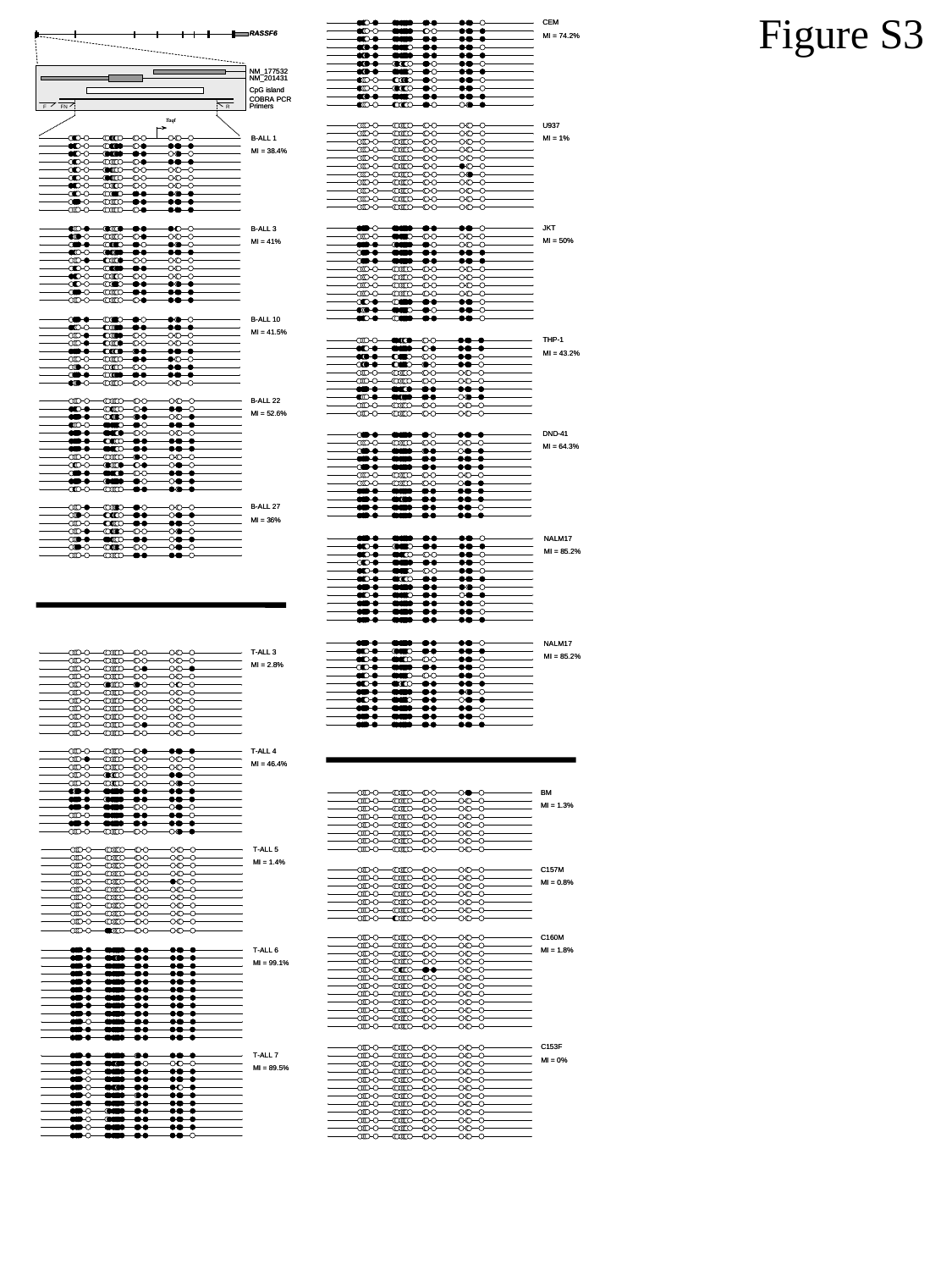

Figure S3

## Slide 4
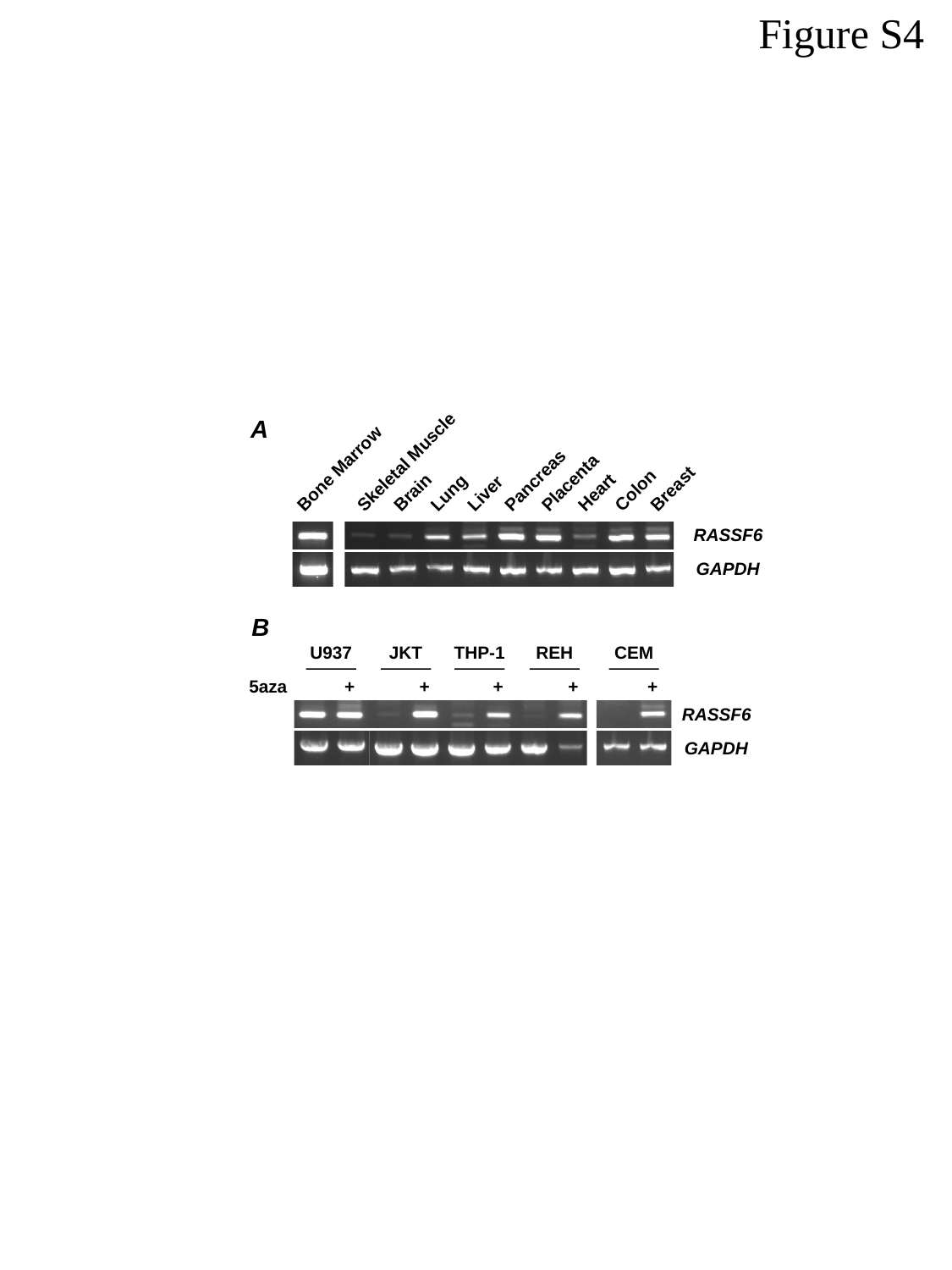

Figure S4
Bone Marrow
Skeletal Muscle
Brain
Lung
Liver
Pancreas
Placenta
Heart
Colon
Breast
RASSF6
GAPDH
A
B
U937
JKT
THP-1
REH
CEM
5aza
+
+
+
+
+
RASSF6
GAPDH

## Slide 5
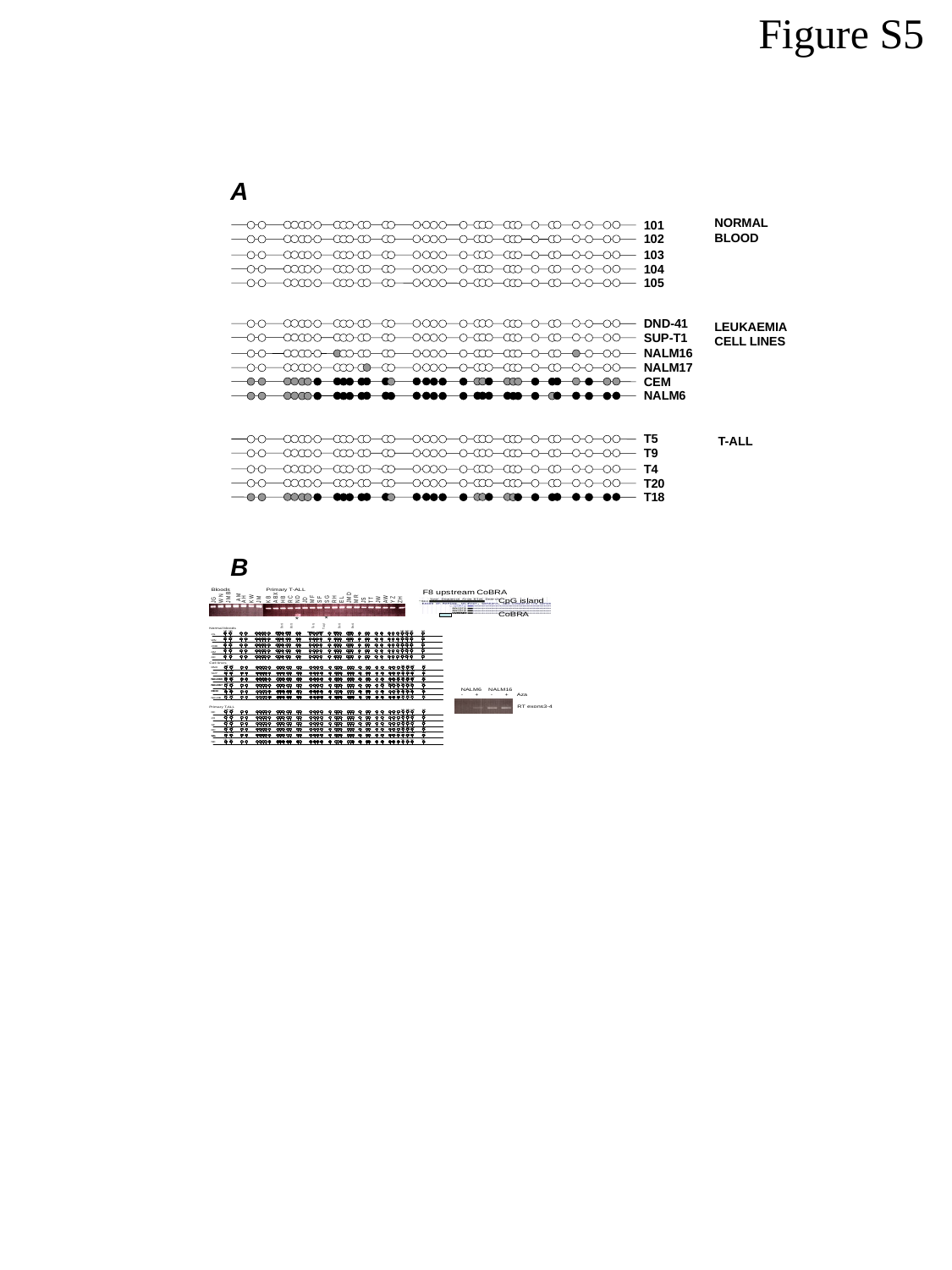

Figure S5
A
NORMAL BLOOD
101
102
103
104
105
DND-41
LEUKAEMIA CELL LINES
SUP-T1
NALM16
NALM17
CEM
NALM6
T5
T-ALL
T9
T4
T20
T18
B
