# Supplementary material for: The novel RASSF6 and RASSF10 candidate tumour suppressor genes are frequently epigenetically inactivated in childhood leukaemias
Source: Mol Cancer. 2009 Jul 1;8:42. doi: 10.1186/1476-4598-8-42 (PMC2711046; doi:10.1186/1476-4598-8-42)
Supplement: Additional file 2 — RASSF6 methylation indexes and PCR primers used within this study. The data provided summarise RASSF6 methylation index data in cell lines, primary tumour and control samples (Table S1). PCR primers used in this study are also listed (Table S2). Table S1: A summary of the methylation index of the RASSF6 CpG island in leukaemia cell lines, B-ALL, T-ALL and normal blood and bone marrow control samples. Table S2: MSP and COBRA primer sequences used within this study. [file 1476-4598-8-42-S2.doc]

**Table S1:** A summary of the methylation index of the *RASSF6* CpG island in leukaemia cell lines, B-ALL, T-ALL and normal blood and bone marrow control samples.

| **TISSUE/TUMOUR TYPE** | **TISSUE/TUMOUR SAMPLE** | **METHYLATION INDEX** |
| --- | --- | --- |
| Leukaemia cell lines | DND-41 | 64.3% |
|  | CEM | 74.2% |
|  | U937 | 1% |
|  | JKT | 50% |
|  | TALL-1 | 80% |
|  | NALM1 | 69.5% |
|  | NALM6 | 64.7% |
|  | NALM17 | 85.2% |
|  | THP-1 | 43.2% |
| Childhood B-ALL | B-ALL 1 | 38.4% |
|  | B-ALL 3 | 41% |
|  | B-ALL 1 | 41.5% |
|  | B-ALL12 | 25.1% |
|  | B-ALL 22 | 52.6% |
|  | B-ALL27 | 36% |
| Childhood T-ALL | T-ALL 3 | 2.8% |
|  | T-ALL 4 | 46.4% |
|  | T-ALL 5 | 1.4% |
|  | T-ALL 6 | 99.1% |
|  | T-ALL 7 | 89.5% |
|  | T-ALL9 | 0% |
| Childhood Pre-B-ALL | Pre-B-ALL | 5.7% |
| Normal Blood | C153F | 0% |
|  | C157M | 0.8% |
|  | C160M | 1.8% |
|  | C170F | 0% |
|  | C189M | 1% |
|  | C194F | 0.5% |
| Normal Bone Marrow | BM | 1.3% |

**Table S2: MSP and COBRA primer sequences used within this study**

| **GENE** | **SYNONYMS** | **ANALYSIS** | **PRIMERS** | **REFERENCE** |
| --- | --- | --- | --- | --- |
| ***RASSF1A*** | - | MSP | MSP F 5’-CGAGAGCGCGTTTAGTTTCGTT -3’  MSP F 5’-CGATTAAACCCGTACTTCGCTAA -3’  USP F 5’-GGGGGTTTTGTGAGAGTGTGTTT -3’  USP F 5’-CCCAATTAAACCCATACTTCACTAA -3’ | Hesson et al., 2004 |
| ***RASSF2*** | - | MSP | MSP F 5’-GTTCGTCGTCGTTTTTTAGGCG -3’  MSP F 5’-AAAAACCAACGACCCCCGCG -3’  USP F 5’-AGTTTGTTGTTGTTTTTTAGGTGG -3’  USP F 5’-AAAAAACCAACAACCCCCACA -3’ | Hesson et al., 2005 |
| ***RASSF3*** | - | COBRA | F 5’-TTGAGGAAGYGATTYGAGTATAGTTTTAGT -3’  R 5’-CAAAAACACRTAAAAACAAAAAACRCRAACTAA –3’  RN 5’-CCACTCACTTATTAACCRAAACRAAACTTA -3’ | Hesson et al., 2004 |
| ***RASSF4*** | *AD037* | COBRA | F 5’-AGGATAYGATATATGTAGTGGTTTTTGGATT -3’  R 5’-ATTATAACCCCTAAATTACTTAACAAAAATACCAAA -3’ | Eckfeld et al., 2004 |
| ***RASSF5A*** | *NORE1A* | COBRA | F 5’-TAGAAATGYGTTTTTTGAGTTGTATTGTAT -3’  R 5’-ATAACTCRAACCCRCTCAAACTCTATAAA -3’  FN 5’-GTTTTAGGTGAAGAYGTTTTTAAATTTATA -3’ | Hesson et al., 2004 |
| ***RASSF6*** | - | COBRA | F 5’-GTATAGGGAGTGGTTTAGGTTTTTTGATAT -3’  R 5’-ATCCCCATTTTTTACCTATTATTCACACTATA -3’  FN 5’-TTGTTGTGGYGAAAAGGAGAAATAATTAATAGT -3’ | This study |
| ***RASSF7*** | *HRC1* | COBRA | F 5’-TTTAGGAGYGGGGTTAGATATTTATTT-3’  R 5’-AACATCRCRAAAAACTCTCCCCAATTAAAA-3’  FN 5’-GGGAGGGGYGTGATAGAGGTAGTTAA-3’ | This study |
| ***RASSF8*** | *HOJ, C12ORF2* | COBRA | F 5’-TTTTATAATGTAGYGTTGGYGTTTTAGTTT-3’  R 5’-CRAAACTCRACRAAACTAAACRAAAAACT -3’  RN 5’-ACTCRACRAAACTAAACRAAAAACTAAAC -3’ | This study |
| ***RASSF10*** | *LOC644943* | COBRA | F 5’-TTGTTTTTGTTGTTTTYGTYGTTTTAGTAGATT-3’  R 5’-CRATTAAACTTAACCAATTTACRAAAAACCTTA-3’  FN 5’-GTGTGGATTTGTTAGGAAGAGAAGT-3’ | This study |
